# A Novel Highly Sensitive Electrochemical Nitrite Sensor Based on a AuNPs/CS/Ti_3_C_2_ Nanocomposite

**DOI:** 10.3390/nano12030397

**Published:** 2022-01-26

**Authors:** Tan Wang, Xianbao Xu, Cong Wang, Zhen Li, Daoliang Li

**Affiliations:** 1 National Innovation Center for Digital Fishery, China Agricultural University, Beijing 100083, China; Tan.wang@cau.edu.cn (T.W.); xianbao_xu@cau.edu.cn (X.X.); ndfic_wc@cau.edu.cn (C.W.); leezn2009@163.com (Z.L.); 2 College of Information and Electrical Engineering, China Agricultural University, Beijing 100083, China; 3 Beijing Engineering and Technology Research Center for Internet of Things in Agriculture, China Agricultural University, Beijing 100083, China; 4 Key Laboratory of Agricultural Information Acquisition Technology, Ministry of Agriculture, China Agricultural University, Beijing 100083, China

**Keywords:** nitrite detection, electrodeposition, nanocomposite, modified electrode

## Abstract

Nitrite is common inorganic poison, which widely exists in various water bodies and seriously endangers human health. Therefore, it is very necessary to develop a fast and online method for the detection of nitrite. In this paper, we prepared an electrochemical sensor for highly sensitive and selective detection of nitrite, based on AuNPs/CS/MXene nanocomposite. The characterization of the nanocomposite was demonstrated by scanning electron microscopy (SEM), a transmission electron microscope (TEM), energy dispersive X-ray spectroscopy (EDX), X-ray diffraction (XRD), cyclic voltammetry (CV), and electrochemical impedance spectroscopy (EIS). Under the optimized conditions, the fabricated electrode showed good performance with the linear range of 0.5–335.5 μM and 335.5–3355 μM, the limit of detection is 69 nM, and the sensitivity is 517.8 and 403.2 μA mM^−1^ cm^−2^. The fabricated sensors also show good anti-interference ability, repeatability, and stability, and have the potential for application in real samples.

## 1. Introduction

Nitrite is a hazardous inorganic pollutant and is commonly found in food, water resources, and agriculture [1]. The high concentration of nitrite in water mainly comes from industrial wastewater discharge and excessive use of agricultural nitrogen fertilizer, which can directly affect water quality, causing water eutrophication, resulting in bacterial breeding and fish death [2,3,4]. Furthermore, a high level of nitrite causes the production of methemoglobin and reduces the oxygen-carrying capacity of red blood cells, which leads to cancer [5]. The World Health Organization (WHO) states that the concentration of nitrite in drinking water should be below 3 mg/L [6]. Therefore, it is very important to develop a method for sensitive, fast, and online detection of nitrite.

At present, some detection approaches for nitrite have been reported, such as Raman spectroscopy [7], fluorescence spectroscopy [8], chromatography [9,10], spectrophotometry [11], chemiluminescence [12], and electrochemical sensors [13,14]. Among them, the electrochemical method possesses many advantageous features of easy operation, rapid response, low cost, and online access [15]. To promote the selectivity and reduce the overpotential of electrochemical sensors, modifying sensing nanomaterials on the working electrode is an effective approach, which has been widely reported upon [16].

Currently, a variety of functional materials have been reported to selectively detect nitrite [17,18,19]. MXenes, composed of metal carbides and metal nitrides, are emerging 2D lamellar functional nanomaterials, which have tunable surface chemistry, hydrophilic surfaces, good electrical conductivity, and good biocompatibility [20,21,22]. However, the interlayer aggregation of MXene impedes electronic transmission and limits its application in electrochemical sensors [20]. Researchers found that MXene nanosheets could be potential supporting materials due to the large surface area, and other nanomaterials used in the decoration of MXene sheets can effectively solve the interlayer aggregation and application in electrochemical sensors [23,24]. Chitosan (CS) is one of the natural amino polysaccharides which have an excellent film-forming ability, and are cost effective, environmentally friendly, and biocompatible [25,26]. Although CS cannot conduct electricity, it is often used in electrochemical sensors because of its excellent property of biocompatibility and film-forming ability. Au nanoparticles have good electrocatalytic capability, electrical conductivity, and biocompatibility, and are widely used for electrochemical sensors. For example, Mo et al. reported a nitrite electrochemical sensor based on AuNPs/graphene/CS, which shows good electrocatalytic activity high sensitivity [27]. Han et al. proposed detection of nitrite based on rose-like AuNPs/MoS_2_/graphene composite. Feng et al. prepared a Au@Carbon quantum dots–MXene nanocomposite for sensitive detection of nitrite [28]. These materials show their unique advantages in the field of electrochemistry, and, to the best of our knowledge, these AuNPs integrated with CS and Ti_3_C_2_ have not yet been reported on in use for an electrochemical nitrite sensor.

In this work, we synthesized a AuNPs/CS/MXene nanocomposite by a facile electrodeposition method. Compared with other methods, electrodeposition is not only a green, fast, and simple method, but can also effectively control the size of synthetic gold nanoparticles. We also optimized the synthesis conditions of nanomaterials and the pH of the detection solution. Due to the synergy of these components, the novel electrochemical sensor, based on AuNPs/CS/MXene nanocomposites, exhibited excellent electrochemical and catalytic properties for sensing nitrite. Furthermore, the prepared electrode also showed good selectivity and stability, and has potential for applications in practical samples.

## 2. Materials and Methods

### 2.1. Chemical Reagents and Materials

Nitrite powder (AR, 99%), HAuCl_4_ (98%), Na_2_HPO_4_ (99.99%), NaH_2_PO_4_ (99.9%), NaNO_3_ (AR, 99%), K_2_SO_4_ (AR, 99%), K_2_CO_3_ (AR, 99%), NHCl (AR, 99.5%), Na_2_SO_3_ (99.9%), Cu(NO_3_)_2_ (AR, 99%), KCl (AR, 99.5%), acetic acid (AR, 99.5%), and chitosan powder (CS, deacetylation, 95%) were purchased from Macklin Chemical Reagent Company (Shanghai, China). Ti_3_AlC_2_ power was purchased from XF NANO, INC (Nanjing, China). HF (AR, 40%), H_3_PO_4_ (85%), HNO_3_ (68%), and absolute ethanol (95%) were purchased from China Agricultural University (Beijing, China). Ultrapure water (18.25 MΩ) was used throughout the experiment and prepared by the laboratory equipment. All of the chemical reagents were analytical grade and were used without further purification.

The PBS (0.1 M) buffer was composed of Na_2_HPO_4_ solution (0.1 M) and NaH_2_PO_4_ solution (0.1 M), the pH value of PBS buffer was adjusted by changing the mixture ratio of components and adding H_3_PO_4_ (0.1 M) or NaOH (0.1 M). CS solution (0.4 wt%) was prepared from CS powder, acetic acid (1 wt%), and deionized water.

### 2.2. Instruments

The conventional three-electrode system was used for the electrochemical experiments. The modified glass carbon electrode (3 mm) was used as a working electrode, a saturated Ag/AgCl was used as a reference electrode, and a platinum wire (1 mm) was used as the auxiliary electrode. All electrodes were obtained from Aida Heng-sheng Technology Development Co., Ltd. (Aida, Tianjin, China). Cyclic voltammograms (CVs) (Gamry 600+, Warminster, PA, USA) and amperometric (i-t) (Gamry 600+, Warminster, PA, USA) measurements were performed by the Gamry 600+ (Warminster, PA, USA) electrochemical workstation.

For AuNPs/CS/MXene characterization, scanning electron microscopy (SEM) and X-ray energy-dispersive spectrometry (EDS) were recorded by Hitachi SU8020 (Hitachi, Tokyo, Japan). The transmission electron microscopy (TEM) images and X-ray diffraction (XRD) data were obtained by Tecnai G2 F30 (FEI, Hillsboro, OR, USA) and Bruker D8-Advance X-ray diffractometer (Bruker, Karlsruhe, Germany).

### 2.3. Preparation of MXene and CS/Mxene

The MXene powder was prepared by our last work [29]. A measure of 0.4 g CS powder was added into 1 mL Acetic acid and then added ultrapure water until 100 g, and was stirred for 2 h to obtain CS solution (0.4 wt%, pH = 4.05). A measure of 50 mg MXene powder was added into 10 mL ultrapure water, and was stirred for 30 min to obtain MXene solution (5 mg/L).

CS/MXene composite materials were synthesis by CS solution and MXene solution were mixed and continuously stirred for 2 h. To study the optimal CS/MXene composite materials, the mixing ratio of CS and MXene was 4:1, 3:1, 2:1, 1:1, 1:2, 1:3, and 1:4, the prepared composite materials were stored in the refrigerator at 4 °C.

### 2.4. Preparation AuNPs/CS/MXene Modified Glass Carbon Electrodes

The bare glass carbon electrode (GCE) was polished with 0.3 μm and 0.05 μm Al_2_O_3_ slurries and washed with HNO_3_, absolute ethanol, and deionized water, respectively. A measure of 5 μL MXene and CS/MXene solution was dropped as coating on the bare electrode surface and dried at room temperature to obtain CS/MXene/GCE and Mxene/GCE.

AuNPs/CS/MXene/GCE was obtained by the electro-reduction method. The prepared CS/MXene/GCE was immersed in PBS (0.1 M, pH = 7) containing 5 mM HAuCl_4_, and reduced by cyclic voltammetry with the potential was set to −1–0.4 V for 10 cycles, the gold nanoparticles (AuNPs) were deposited on the CS/MXene/GCE surface to obtain AuNPs/CS/MXene/GCE. AuNPs/MXene/GCE was prepared by the same method.

### 2.5. Modified Electrodes Detection Nitrite

All the electrochemical tests were carried out by our electrochemical workstation with a standard 3-electrode system, the working electrode was modified GCE and the counter electrode used a platinum wire (1 mm) electrode, with Ag/AgCl electrode as the reference electrode. Cyclic voltammetry (CV, −0.2–0.6 V) and electrochemical impedance spectroscopy (EIS, 0.1–10^5^ HZ) were used to test the electrochemical characteristic of modified GCE in 0.1 M KCl contain 10 mM K_3_[Fe(CN)_6_], the electrochemical response of modified electrode towards nitrite by CV (0.4–1.2 V, 50 mV/s). Chronoamperometry was performed under the potential of +0.8 V.

## 3. Results

### 3.1. Structure and Surface Morphology of AuNPs/CS/MXene

**Figure 1:**
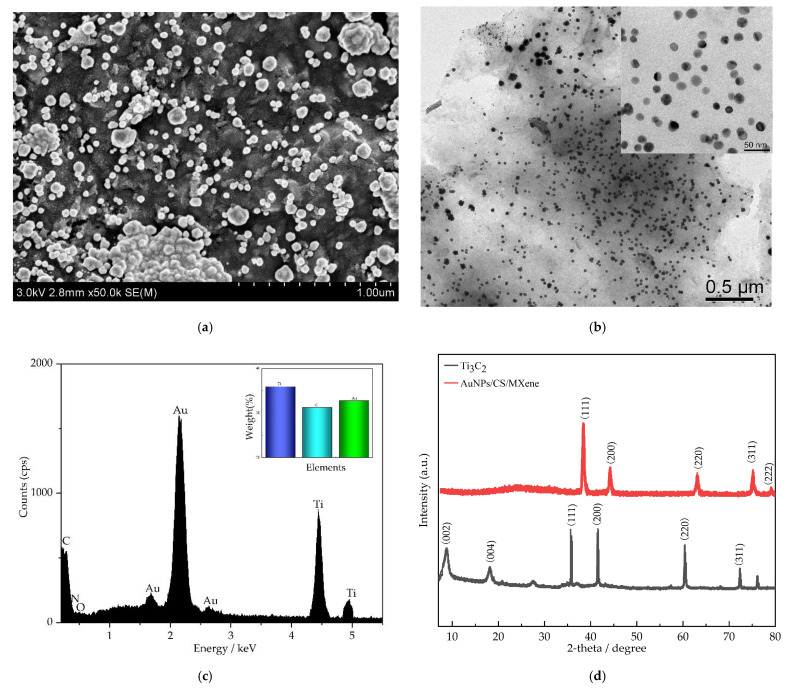
SEM images (**a**), TEM images (**b**), EDX (**c**), and XRD (**d**) of AuNPs/CS/MXene.

Figure 1 shows the characterization of AuNPs/CS/MXene nanocomposite by SEM, TEM, EDS, and XRD. The SEM image of the nanocomposite is given in Figure 1a, showing the AuNPs/CS/MXene nanocomposite. Some round particles with uneven appearances were dispersed in the surface of the nanocomposite, the round particles are AuNPs, showing electrodeposition by CVs. In Figure 1b, the TEM image of AuNPs/CS/MXene nanocomposite shows MXene has between one and a few layers, with a very thin and highly transparent morphology, the insert image shows that the size of AuNPs is about 20 nm. The EDX of AuNPs/CS/MXene was tested as shown in Figure 1c, which showed signals of C, O, N, Au, and Ti, and the weight percentage shown in the Figure 1c insert. The XRD patterns of Ti_3_C_2_ and AuNPs/CS/MXene are shown in Figure 1d; the pattern of Ti_3_C_2_ reveals several characteristic diffraction peaks at 2*θ* 8.63° (002), 18.15° (004), 35.74° (111), 41.54° (200), 60.45° (220), and 72.43° (311); the AuNPs/CS/MXene shows five peaks, appearing at 38.36° (111), 44.43° (200), 63.21° (220), 75.28° (311), and 79.22° (222), indicating that the (111) lattice plane of Au is the primary orientation.

### 3.2. Electrochemical Characterization of the Sensor

**Figure 2:**
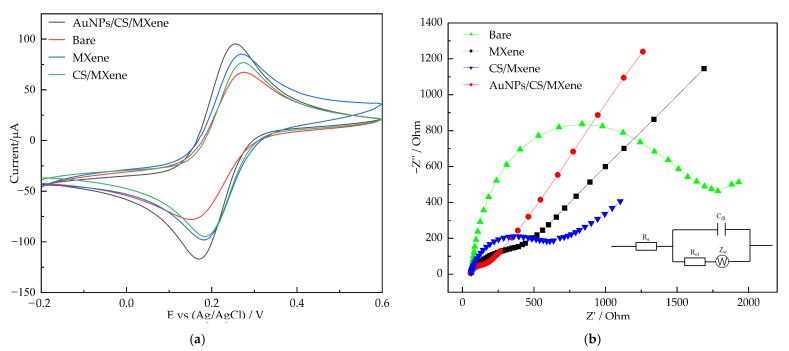
CV curves (**a**) and EIS (**b**) of bare GCE, CS/MXene/GCE, MXene/GCE, and AuNPs/CS/MXene/GCE in 0.1M KCl containing 10 mM of K_3_[Fe(CN)_6_].

The electrochemical characterization of AuNPs/CS/MXene/GCE was investigated by CV and EIS in 0.1 M KCl solution and contained 10 mM K_3_[Fe(CN)_6_]. This is an effective method to determine the electron transfer properties of modified electrodes. Figure 2a showed the comparative CV curves of bare GCE, MXene/GCE, CS/MXene/GCE, and AuNPs/CS/MXene in the solution containing 10 mM K_3_[Fe(CN)_6_] and 0.1 M KCl. It shows that the oxidation peak current of the modified electrode is significantly higher than that of the bare electrode. Among them, the peak current of AuNPs/CS/MXene/GCE is the highest, which means that the combination of AuNPs, CS, and MXene as composite materials have high electrocatalytic activity for nitrite oxidation.

Figure 2b demonstrates the EIS (Nyquist plots) of bare GCE, MXene/GCE, CS/MXene/GCE, and AuNPs/CS/MXene/GCE in 0.1 M KCl containing 10 mM K_3_[Fe(CN)_6_]. Randles equivalent circuit model has been used to fit the experimental data (inset to Figure 2b), in which R_ct_, R_s_, C_dl_, and Z_w_ depicted charge transfer resistance, electrolyte resistance, double-layer capacitance, and Warburg element, respectively. In the Nyquist plots, the diameter of the semicircle represents the electron transfer resistance (R_et_). Compared with bare GCE, modified electrodes (CS/MXene/GCE, MXene/GCE, and AuNPs/CS/MXene/GCE) show the semicircle with a smaller diameter, which means that the electron transfer resistance is reduced. Among them, EIS of AuNPs/CS/MXene/GCE exhibited the smallest semicircle with the R_et_ values of 70.7 Ω, owing to the synergistic effect between AuNPs, CS, and MXene, which revealed the most excellent conducting properties.

### 3.3. Electrocatalytic Response of Different Modified GCEs towards Nitrite

**Figure 3:**
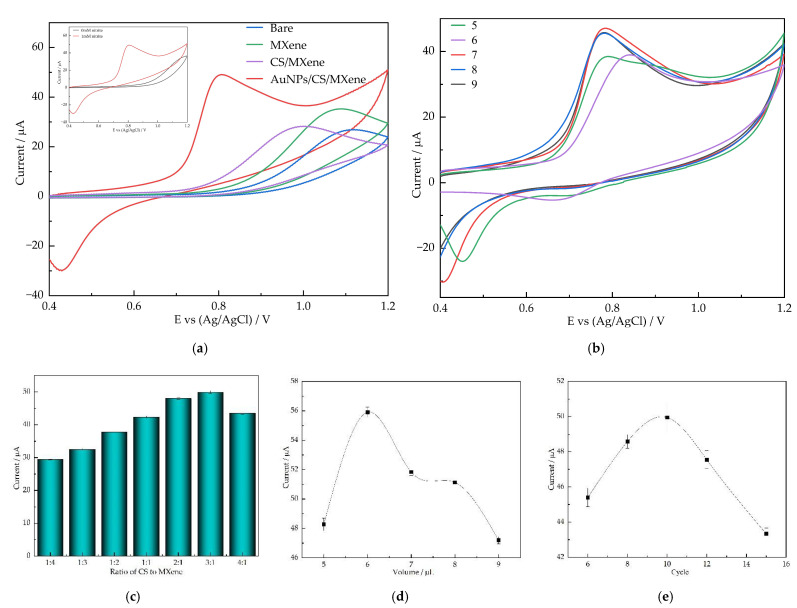
(**a**) CV curves of different electrode in 0.1 M PBS (pH 7) containing 1 mM nitrite. Inset shows CV curves of AuNPs/CS/MXene/GCE in the absence and presence of 1 mM nitrite. (**b**) Peak current of the AuNPs/CS/MXene/GCE in 0.1 M PBS containing 1 mM nitrite with different pH values. Peak current of the AuNPs/CS/MXene/GCE in PBS containing 1 mM nitrite with different ratio of CS to MXene (**c**), different volume of the modifier (**d**), and the cycle of CV electrodeposition (**e**).

The electrochemical property of the modified electrode towards nitrite oxidation was investigated by CV experiments. Figure 3a show the comparative CV curves (0.4–1.2 V, 50 mV/s) of bare GCE, MXene/GCE, CS/MXene/GCE, and AuNPS/CS/MXene/GCE for the oxidation of 1 mM nitrite in 0.1 M PBS (pH 7). The CV curves of the bare GCE, MXene/GCE, CS/MXene/GCE, and AuNPs/CS/MXene/GCE show that the oxidation peak occurred at the potential +1.12, +1.09, +1 V, and 0.8 V, respectively, and the oxidation current of these electrodes is 26.9, 35.3, and 28.4 μA, respectively. Compared with bare GCE, the oxidation current of the modified electrode was enhanced, and the overcurrent was decreased. Notably, the nitrite oxidation peak current was 2.1-fold enhanced at AuNPs/CS/MXene/GCE compared with those at bare GCE, and was better than MXene/GCE and CS/MXene/GCE. This is due to the synergic effect between individual components, which facilitated the AuNPs/CS/MXene nanocomposite to have good electro-catalytic performance towards the oxidation of nitrite.

To promote the electrocatalytic performance of the modified electrode, we studied the synthesis of nanomaterials under different conditions. The optimized experimental conditions include the ratio of CS to MXene, the amount of CS/MXene composite, the number of cycles of electrodeposition, and the pH of nitrite solution (*n* = 5). Firstly, the effects of pH were investigated using CV in 0.1 M PBS solution containing 1 mM nitrite at different pH values (pH = 5–9). As shown in Figure 3b, the nitrite oxidation current of AuNPs/CS/MXene/GCE increased with increasing pH from 5–7 and decreased from pH 7–9. Thus, indicated that the modified electrode showed the best electrochemical activity at pH 7. The following experiments are controlled to a pH of 7. The ratio of CS to MXene has affected the nitrite oxidation current, as shown in Figure 3c, the maximum oxidation peak current occurs when the ratio of CS to MXene is 3:1. Additionally, then, the effects of the amount of CS/MXene composite (CS:MXene = v3:1) on nitrite oxidation current were investigated (Figure 3d). The results show that a better catalytic effect can be achieved by drop coating 6 μL of CS/MXene. Figure 3e shows the nitrite response current of the different number of cycles of CV electrodeposition. The results show that the nanomaterials prepared by 10 cycles of CV electrodeposition have better properties than those with other number cycles. Thus, the following experiments were carried out under the above-optimized conditions.

**Figure 4:**
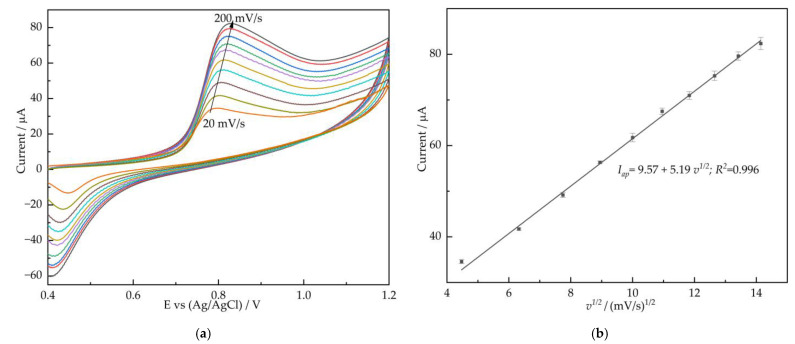
(**a**) The CVs of AuNPs/CS/MXene/GCE in 0.1 M PBS (pH = 7.4) containing 1 mM nitrite at different scan rates (20–200 mV/s); (**b**) plot of anodic peak current and cathode peak current of nitrite versus the square root of scan rate.

The electro-catalytic oxidation reaction mechanism of nitrite on AuNPs/CS/MXene/GCE is discussed, the CV experiments at scan rates from 20 to 200 mV/s were recorded. Figure 4a shows the anodic peak current (*I_ap_*) increased with the increase in scan rates, while the peak potential shifted positively. Figure 4b shows the linear relationship of *I_ap_* versus *v^1/2^*, indicated by a diffusion-controlled process, and the linearization equation were *I_ap_* = 9.57 + 5.19*v^1/2^* (*R*^2^ = 0.996). The electrocatalytic response mechanism of nitrite to the AuNPs/CS/MXene/GCE can be expressed as follows:

NO_2_^−^ + H_2_O − 2e^–^ → NO_3_^−^ + 2H^+^

### 3.4. Amperometry

**Figure 5:**
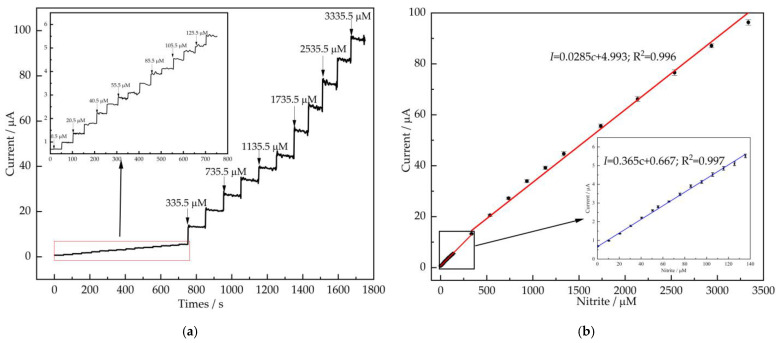
(**a**) Amperometric profiles of AuNPs/CS/MXene/GCE to the successive additions of nitrite (0.5–3335.5 μM) in 0.1 M PBS (pH = 7) at the applied potential of +0.8 V with continuous stirring (1200 r/min), the inset is the response of AuNPs/CS/MXene/GCE to low-concentration nitrite; (**b**) the calibration curve between the response currents and concentrations of nitrite, the insert is the calibration curve at low-concentration nitrite.

To evaluate the sensing application of modified electrode, the amperometry is generally tested for the current response of nitrite concentration by measuring the current response at a fixed potential upon addition of analyte. Figure 5a exhibited the amperometric response of the AuNPs/CS/MXene/GCE at +0.8 V in the 0.1 M PBS (pH = 7) solution addition of nitrite with continuous stirring. It was seen that a quick and stable current response after the addition of nitrite concentration and reached the steady state value within 5 s.

Figure 5b shows the linearity between the current response and the concentration of nitrite. The calibration plot shows 2 linear ranges, the concentration of nitrite from 0.5 μM to 355.5 μM, a linear equation is *I* = 0.0366*c* + 0.667 (R^2^ = 0.997), the sensitivity is 517.8 μA mM^−1^ cm^−2^, and the limit of detection (LOD = 3SD/S) was calculated to be 69.2 nM, respectively. The concentration of nitrite from 355.5 μM–3.35 mM show the linear equation is *I* = 0.0285*c* + 4.993 (*R*^2^ = 0.996), the sensitivity is determined to be 403.2 μA mM^−1^ cm^−2^. The characteristics of the reported sensors are shown in Table 1. Our work shows better performance than most of the reported nitrite sensors, which indicated that the AuNPs/CS/MXene/GCE was an alternative platform for the detection of nitrite.

### 3.5. Selectivity of the Electrode

**Figure 6:**
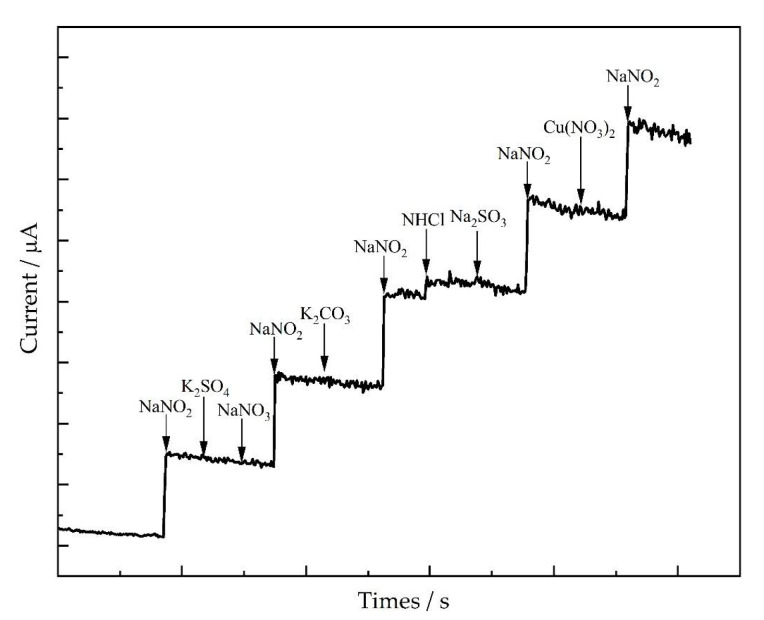
Interference studies for AuNPs/CS/MXene/GCE with the addition of 10 μM nitrite and 1000 μM various interferences by amperometry, applied potential: +0.8 V; rpm: 1200.

To investigate the selectivity of AuNPs/CS/MXene/GCE to nitrite, a chronoamperometry method was used at +0.8 V with continuous stirring (1000 rpm) in 0.1 M PBS (pH = 7). In this experiment, the uses of 1000 μM NHCl, K_2_SO_4_, NaNO_3_, Na_2_SO_3_, K_2_CO_3_, and Cu(NO_3_)_2_, as the interferences, were investigated. As shown in Figure 6, with the addition of 10 μM nitrite, the response current increased significantly, while the current cannot change obviously by adding a 100-fold interfering substance. Thus, the AuNPs/CS/MXene/GCE was demonstrated to have good selectivity to nitrite.

### 3.6. Repeatability and Stability

**Figure 7:**
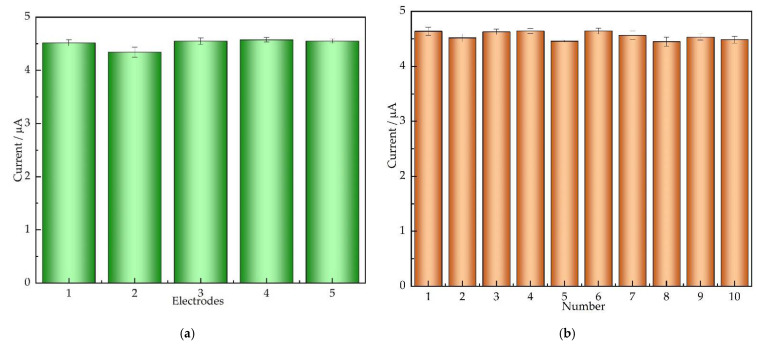
The current response of 5 modified electrodes (**a**) and 10 successive measurements (**b**) in 0.1 M PBS (pH = 7) containing 100 μM nitrite.

The reproducibility of AuNPs/CS/MXene/GCE was determined by CVs in 0.1 M PBS (pH = 7) containing 100 μM nitrite, as shown in Figure 7. The relative standard deviation (RSD) of the current response among five AuNPs/CS/MXene/GCE prepared in the same conditions was 2.08%, and the RSD value was 1.72% for 10 successive measurements, which demonstrates the AuNPs/CS/MXene/GCE has high repeatability. Moreover, the modified electrode stored at 4 °C for one month, the current response was remained 97.1% of its original response in 100 μM nitrite, indicating a long service life.

### 3.7. Real Sample Analysis

The practical application of the sensor to detect nitrite in the real sample was assessed by the standard addition method. Real water samples were filtered by a 0.22 μm membrane, use of ample preparation refers to the work by Majidi [36]. All samples had PBS (pH = 7) as the supporting electrolyte, quantitative nitrite was added to all treated water samples and analyzed using a AuNPs/CS/MXene nanocomposite-modified electrode by amperometry. As shown in Table 2, the values measured by this method were compared with those measured by the addition and UV-Vis method. Additionally, the *t*-test was used for the statistical comparison of the obtained results. The t value at a confidence level of 95% was 3.18 (α = 0.05) and the obtained t values were smaller than the critical ones, revealing that there was no difference between the obtained results, which implies the modified electrode has great potential for monitoring nitrite in real samples.

## 4. Conclusions

In this work, we presented a novel electrochemical sensor for determining nitrite, based on a AuNPs/CS/MXene nanocomposite. The AuNPs/CS/MXene nanocomposite was constructed by a facile electrodeposition process, it exhibited excellent electro-catalytic activity towards the analysis of nitrite. During this study, the experimental conditions were optimized and the nitrite sensor showed good performance in sensing nitrite. More important, the selectivity, repeatability, and stability of the prepared sensor were verified, and it has the potential for application in actual samples. 

## Figures and Tables

**Table 1:** Performance comparison of different modified electrodes for nitrite determination.

Electrode Materials	Linear Range (μM)	LOD (μM)	Sensitivity (μA mM^−1^ cm^−2^)	Ref.
AuNPs/MoS_2_/GN	5–5000	1	N/A	[1]
MWCNTs/PPy-C	5–9500	3.06	117.1	[30]
MWCNTs/Co-MOFs	80–1160	18.8	10	[31]
Pt-Cu/GO	0.2–9000	3	139.9	[32]
rGO/ZnO/Nafion	20–520	1.36	375.4	[33]
PEDOT/PEDOT-SH/Au	0.15–1000, 1000–16,000	0.051	301 133	[34]
CeO_2_-SnO_2_/Pd	0.36–2200	0.1	652.95	[35]
AuNPs/CS/MXene	0.5–335.5 335.5–3300	0.069	517.8 403.2	This work

**Table 2:** Determination of nitrite in water samples (*n* = 3).

Sample	Added (μM)	This Method (μM)	UV-Vis Method (μM)	RSD (%)	*t*-Test
Tap water	20	20.59	20.29	3.4	1.77
River water	20	20.78	20.61	2.3	1.14
Sausage	20	20.59	20.83	3.5	0.95

## Data Availability

The data presented in this study are available on request from the corresponding author.
